# Evaluating the Contributions of Dynamic Flow to Freezing of Gait in Parkinson's Disease

**DOI:** 10.4061/2010/732508

**Published:** 2010-03-24

**Authors:** Chad A. Lebold, Q. J. Almeida

**Affiliations:** Movement Disorders Research and Rehabilitation Centre, Wilfrid Laurier University, 75 University Avenue West, Waterloo, Ontario, Canada N2L 3C5

## Abstract

Although visual cues can improve gait in Parkinson's disease (PD), their underlying mechanism is poorly understood. Previous research suggests that cues contribute optical flow that is essential to elicit gait improvement. The present study manipulated how optic flow was provided, and how this might influence freezing of gait (FOG) in PD. Therefore, three groups; 15 PD FOG, 16 PD non-FOG, and 16 healthy controls were tested in 3 narrow doorway conditions; baseline (Narrow), ground lines (Ground), and laser (Laser). Step length indicated that the PD FOG group was only able to improve with ground lines, while the laser increased gait variability and double support time. These results suggest that optic flow in itself is not enough to elicit gait improvement in PD. When PD patients use visual cues, gait becomes less automatically controlled and hence preplanned conscious control may be an important factor contributing to gait improvement.

## 1. Introduction

An impaired gait pattern consisting of a smaller than normal step length and decreased gait velocity, leading to an increased risk of falls, has been well documented in Parkinson's disease (PD) [1–3]. It has been established that visual cues are a helpful strategy for improving the impaired gait in PD [1, 2, 4–6]. However, while it is clinically well known that visual cues can overcome freezing of gait (FOG), little research has evaluated the underlying mechanism of how visual cues contribute to the prevention of FOG. Visual cues such as ground lines have been argued to improve gait by forcing a more conscious mode of control to regulate gait, hence bypassing the dysfunctional basal ganglia [1]. As such, these results support the hypothesis that increased attentional focus is specifically responsible for improving step length in individuals with PD when using ground lines as a visual step cue. Importantly, an alternative viewpoint has been suggested by Azulay et al. [4, 7] who removed the availability of optic flow using stroboscopic lighting from visual cues. Since PD participants could not improve step length in this condition, the authors concluded that the dynamic flow derived from the visual cues must be essential to elicit gait improvement. Other research has also suggested that the removal of optic information may lead to decreased stride length and slowed gait velocity [8]. Rather than removing optic flow, the primary objective of the present study was to manipulate how optic flow was provided in order to evaluate these hypotheses. Since it is not practical to lay lines of tape on the ground wherever a patient might walk, the current study compared the benefits associated with visual cues taped to the ground to those gained with the use of a portable, motorized laser device that acted as a step cue which closely matched normal optic flow from the environment. 

A secondary objective of this research was to evaluate how the manipulation of visual cue availability would influence FOG, one of the most severe gait impairments occurring in PD [9]. FOG is characterized by a sudden inability to initiate or continue walking, especially while turning; in stressful time-constrained situations; and upon entrance into and through confined spaces such as doorways [10–14]. Previous research has tested a visual cue device that provided a laser beam projected from the distal end of a walking cane in an attempt to overcome freezing [15]. Unfortunately, results indicated that the device was unable to decrease the number of FOG episodes, however the researchers did not measure any changes to gait that may have indicated potential benefits of using the device [15]. Recent research has demonstrated that increased stride-to-stride variability occurs prior to FOG, as compared to PD patients without FOG [16]. Hausdorff et al. demonstrated that the ability to regulate stride-to-stride timing during gait is severely impaired in FOG patients, as compared to other individuals with PD. Hence, analysis of stride-to-stride variability is an important and useful method of identifying characteristics of gait that are closely linked to freezing, as seen in studies where negotiating of an obstacle or a narrowing of space such as a doorway is used [17, 18]. Hence, the final aim of this experiment was to manipulate the presence and type of visual cue while traveling towards an narrowed space, in order to determine how spatial-temporal aspects of gait might be influenced.

## 2. Methods

### 2.1. Subjects

The study involved 31 participants with PD (15 confirmed to be experiencing FOG at the time of test, 16 absent of FOG) and 16 healthy, age-matched control participants (no significant differences for age, height, UPDRS score, or years since diagnosis between groups, for full details of participant characteristics see Table 1) recruited from a database at the Movement Disorders Research and Rehabilitation Centre at Wilfrid Laurier University in Waterloo, Canada. In this database, participants in the PD FOG group were selected based on their self-report of experiencing freezing, when filling out a questionnaire formatted to represent UPDRS (Section 2). Additionally, a trained clinician confirmed the occurrence of freezing in these patients during an earlier assessment and at the time of test (see Procedures below). 

All patients that were tested had clinically typical PD as confirmed by diagnosis from at least one movement disorder neurologist and were known to be responsive to antiparkinsonian medication. All participants with PD were tested approximately 1 hour after having taken their antiParkinson's medication. However, criteria were used to verify that individuals in the FOG group were experiencing episodes of freezing at the time of test (see Procedures below). Participants in the PD non-FOG group scored at least a 1 (out of 4) on the gait portion of the Unified Parkinson's Disease Rating Scale (UPDRS-Motor section III) by a movement disorder specialist. Sixteen healthy control subjects also participated in the study. These individuals were mostly spouses or relatives of the PD participants.

Subjects were excluded from testing if they had a past history of neurological conditions other than PD, or orthopedic or visual disturbances that severely impaired walking ability. Participants were also excluded if they had been diagnosed with dementia (participants had scored above 24 on the MMSE within the last 6 months) or experienced dyskinesias which would alter their gait pattern. Each participant was informed about the requirements of the study and signed institutionally approved consents, according to the declaration of Helsinki (BMJ 1991; 302: 1194).

### 2.2. Materials

The room used for data collection was a laboratory containing a doorway leading out into an empty hallway. The lighting in both the laboratory and the hallway was maintained at a consistent brightness. Data was collected on a *GAITRite* carpet (*GAITRite*, CIR System, Inc., Clifton, NJ, USA) which is 3.96 m long × 0.79 m wide and contains sensors that send information received from the participants' footsteps to an attached computer. A researcher walked alongside (and slightly behind) the participant at all times for safety of the participant during each trial

### 2.3. Procedure

Before the experiment began, each participant completed a thorough assessment to verify the occurrence of freezing at the time of test (for details see [18]). A modified timed up-and-go test (TUG) was given in which the participant stood up out of a chair, walked through a normal sized doorway, and returned back to a seated position in the chair. This was observed by a movement disorder specialist that confirmed FOG was occurring during the TUG before the normal testing procedure commenced. If the patient did not exhibit FOG during this test, the patient was excluded from testing. 

Subsequently, participants walked the length of the *GAITRite* carpet under three different conditions for five trials each. They began each trial two metres before the start of the *GAITRite* carpet to ensure that the initiation of gait was not recorded. Across all conditions participants traveled through a narrowed (3/4th size) doorway (0.675 m wide × 2.1 m high) which was just wide enough to allow passage without the need to turn or twist their shoulders. Three randomized conditions were examined in this study.

- Baseline doorway condition (Narrow) in which the participant walked normally across the *GAITRite* carpet through the narrowed doorway.
- Ground lines condition (Ground) in which the participant walked across the *GAITRite* carpet while making consecutive heel contacts on ground lines provided by a black overlay placed on top of the entire *GAITRite* carpet through the narrow doorway. This black overlay was made out of a thin cloth material with white horizontal lines 3 cm wide spaced at 65 cm intervals. It was designed as not to impede the normal functioning of any of the sensors in the *GAITRite* carpet.
- Laser condition (Laser) in which the participant walked across the *GAITRite* carpet while making consecutive heel contacts on lines on the ground provided by a motorized laser line device, through the narrow doorway. This device projected a roughly 1 cm wide transverse LED laser line onto the ground ahead of the participant. The initial projection was approximately 65 cm in front of the participant and then the motor rotated the laser line directly towards the participant, therefore traveling in the same direction as regular optic flow that would have been consistent to that obtained from other objects in the environment. The individual was instructed to touch their toe to the laser line when it was at its furthest point. The laser then shut off, rotated back to the initial position, and then appeared again so that participants could continue to walk down the length of the carpet repeating this procedure in sequence with the movement of the laser. The laser device was attached by a belt to each participant's waistline and the constant angle in which the laser was set led to a maximum projection distance that was based on the height of each individual, and therefore an average step length. The laser was set at a fixed speed of one cycle per second, as this was deemed to be an appropriate speed in order to be able to follow the laser as accurately as possible.

### 2.4. Design and Statistical Analysis

The three conditions in this protocol allowed for the analysis of whether additional visual cues in the form of either ground lines or laser lines can possibly prevent the impaired gait experienced by individuals with PD experiencing FOG as they approach a situation that regularly induced FOG episodes. Since previous research has suggested spatial-temporal indicators prior to a freeze occurring [16], only the individuals' gait preceding the doorway was analyzed in order to determine what effect these visual cues have on gait prior to a possible FOG episode.

There were three independent groups in this experiment; individuals with PD experiencing FOG (PD FOG), those with PD experiencing gait abnormalities absent of FOG (PD Non-FOG), and healthy control subjects (Controls). The primary dependant variables analyzed were gait velocity (cm/s), cadence (steps/min), mean step length (cm), base of support (cm), step time (s), and time spent in double support (s). Two measures of step-to-step variability were calculated for each of the spatiotemporal measures: (a) within-trial standard deviation around each individual participant's mean value within a trial was averaged across participants in a given group, and (b) the coefficient of variation (CV) within a trial was calculated based on standard deviation (see (a)) divided by the average value for a given trial, in order to account for variability normalized to the mean. Left and right steps were pooled and results were analyzed by the STATISTICA computerized statistical package using a mixed model 3 groups × 3 conditions × 5 trials ANOVA. In order to determine where the significant differences found in the ANOVA's occurred, Tukey's Honest Significant Difference (HSD) post hoc procedure was employed.

## 3. Results

### 3.1. Velocity

A main effect of group was identified (F(2,44) = 10.12, *P* < .001), and post hoc analysis confirmed that the PD FOG group walked significantly slower (76.49 ± 25.87 cm/s) as compared to the PD non-FOG group (93.04 ± 12.42 cm/s) (*P* < .037) and the Control group (105.56 ±  13.15 cm/s) (*P* < .001). Although there was a 12% difference, the difference between PD non-FOG group and the Control group was not statistically significant.

**Figure 1:**
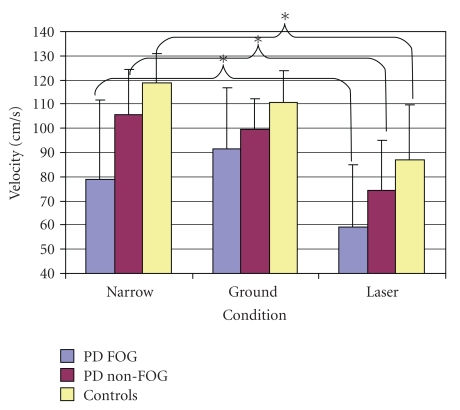
Examination of velocity reveals an interaction of group and condition.

A significant interaction was also found between group and condition (F(4,88) = 2.52, *P* < .0465) (Figure 1). Post hoc analysis determined the existence of a number of significant differences driving this interaction. In contrast to the baseline condition (Narrow), PD FOG significantly decreased their velocity in the laser condition (from 78.91 ± 32.6 cm/s to 59.13 ± 25.98 cm/s) (*P* < .008) but did not significantly alter velocity with ground lines (97.43 ± 25.27 cm/s). In contrast to the baseline (Narrow), PD non-FOG did not increase velocity in the ground line condition (105.59 ± 18.67 cm/s and 99.36 ± 12.89 cm/s, resp.). However, similar to the PD FOG group, the PD non-FOG group experienced a significant decrease in velocity in the laser condition (74.16 ± 20.68 cm/s) (*P* < .001). Healthy control participants displayed the same pattern as observed with PD Non-FOG, as baseline velocity (119.02 ± 11.96 cm/s) was significantly faster than observed when using the laser (86.86 ± 22.98 cm/s) (*P* < .001) and not significantly different from the ground lines condition (110.79 ± 12.83 cm/s). Overall, all participants decreased their velocity when using the laser device.

### 3.2. Step Length

As expected, there was a significant difference in mean step length when comparing the three groups (F(2,44) = 17.48, *P* < .001). Post hoc analysis confirmed that PD FOG (50.95 ± 11.09 cm) took significantly shorter steps than both PD Non-FOG (61.27 ± 5.28 cm) (*P* < .002) and Controls (67.18 ± 5.53 cm) (*P* < .001).

**Figure 2:**
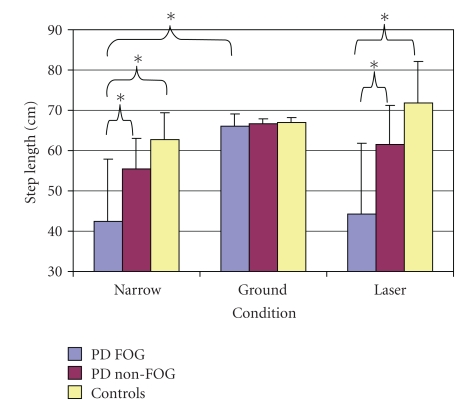
Significant interaction of group and condition in step length.

More importantly, a significant interaction was identified between group and condition (F(4,88) = 14.65, *P* < .001) (Figure 2). Post hoc analysis confirmed a number of key differences. PD FOG (42.53 ± 15.4 cm) had a significantly shorter step length in the baseline condition (Narrow) as compared to PD Non-FOG (55.4 ± 7.72 cm) (*P* < .001) and Controls (62.71 ± 6.6 cm) (*P* < .001). This effect was also found in the laser condition where the PD FOG group step length (44.18 ± 17.68 cm) was again significantly smaller than both PD Non-FOG (61.65 ± 9.47 cm) (*P* < .001) and Controls (71.84 ± 10.29 cm) (*P* < .001). 

Importantly, there was no difference in the step lengths of the three respective groups when they were using ground lines as a visual step cue. PD FOG experienced a significant increase in step length in the ground line condition (66.15 ± 2.86 cm) as compared to both the baseline (Narrow) (*P* < .001) and laser (*P* < .001) conditions. There was also a significant increase in step length observed in PD Non-FOG in the ground condition (66.75 ± 1.07 cm) as compared to the baseline condition (Narrow) (*P* < .001). It is also worth noting that neither PD FOG nor PD Non-FOG revealed any significant improvements in step length with the laser device above the baseline condition (Narrow). It should also be pointed out that healthy controls exhibited an increased step length in the laser condition as compared to the baseline condition (Narrow) (*P* < .015).

### 3.3. Cadence

A main effect of condition was found with regards to cadence, displaying a significantly decreased number of steps per minute in both types of visual cue conditions as compared to baseline (F(2,88) = 98.29, *P* < .001). Also, a significant interaction was identified between group and condition (F(4,88) = 3.85, *P* < .007). All three groups displayed a statistically similar cadence (with regards to condition) except in the ground lines condition where the PD FOG group had a significantly reduced cadence (82.71 ± 20.85 steps/min) as compared to Controls (99.12 ± 10.64 steps/min, *P* < .018).

#### 3.3.1. Step Time

**Figure 3:**
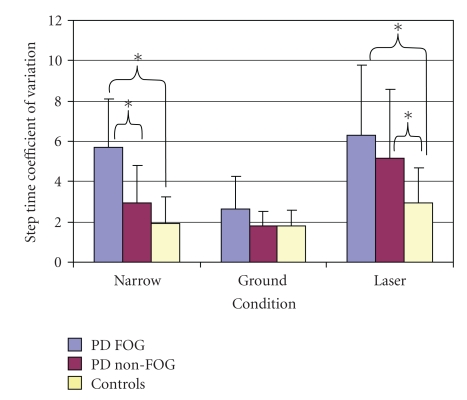
Significant interaction of group and condition in step time coefficient of variation.

Without the data being normalized, the only significant effect with regards to step time was a main effect of condition (F(2,88) = 35.03, *P* < .001). Participants experienced a significantly shorter step time in the baseline condition (Narrow) (0.57 ± 0.23 s) as compared to the ground (0.69 ± 0.16 s) and laser (0.85 ± 0.19 s) conditions. The CV of step time was analyzed and while their was no overall significant main effect of condition, a significant interaction existed between group and condition (F(4,88) = 3.34, *P* < .014) (Figure 3). The PD FOG group was found to have a significantly higher CV of step time in both the baseline condition (Narrow) and laser condition, compared to the PD non-FOG group. Interestingly, only in the ground condition did the three groups exhibit a consistent and improved CV of step time. The laser condition led to an increased CV of step time in the PD FOG group (6.32 ± 3.46, *P* < .001) and PD non-FOG group (5.15 ± 3.4, *P* < .035) as compared to the Controls (2.92 ± 1.79).

### 3.4. Variability of Step Length

**Figure 4:**
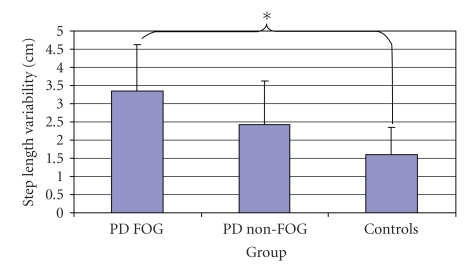
Examination of step length variability reveals a main effect of group.

A main effect of group was significant for within trial step-to-step variability of step length (F(2,44) = 10.01, *P* < .001) (Figure 4). Post hoc analysis revealed that the PD FOG group (3.35 ± 1.28 cm) was significantly more variable as compared to the Control group (1.60 ± 0.74 cm) (*P* < .001). A trend almost reaching significance was also found showing that the step length of the PD FOG group was slightly more variable than the PD non-FOG group (2.43 ± 1.19 cm) (*P* < .062). A main effect of condition was also identified (F(2,88) = 16.62, *P* < .001). 

Post hoc analysis revealed that participants had significantly greater step-to-step variability in the laser (4.02 ± 2.77 cm) as compared to both the baseline condition (Narrow) (1.94 ± 1.54 cm) (*P* < .001) and ground condition (1.46 ± 0.95 cm) (*P* < .001). There were no significant interactions between group and condition identified with regards to within-trial step length variability.

**Figure 5:**
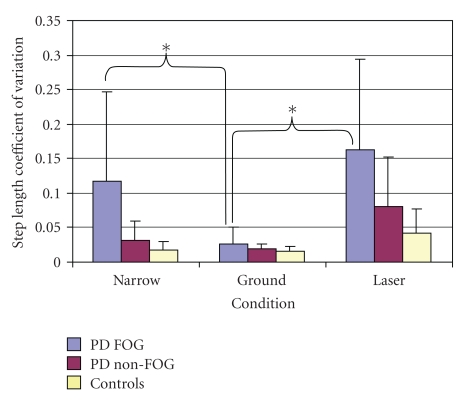
Examination of step length coefficient of variation reveals an interaction of group and condition.

In order to normalize against mean values, the CV of step length was analyzed revealing a significant group by condition interaction (F(4,88) = 4.49, *P* < .003) (Figure 5). Post hoc analysis revealed that both the non-PD FOG and healthy control groups had very low step length variability, across all conditions. Only the PD FOG group showed a significant change in normalized step length variability that was influenced by condition. Interestingly, the only condition that permitted PD FOG to improve normalized step length variability to the level of healthy participants was the ground condition (0.027 ± 0.024), whereas both the Narrow (0.118 ± 0.129, *P* < .002) and laser conditions resulted in a significant increase in normalized step length variability (0.163 ± 0.131, *P* < .001).

### 3.5. Double Support Time

The amount of time that individuals spent in the double support phase of gait cycle differed significantly as indicated by a main effect of group (F(2,44) = 3.76, *P* < .031). Post hoc analysis confirmed that the PD FOG group (0.52 ± 0.46 s) spent significantly more time in double support as compared to Controls (0.27 ± 0.06 s) (*P* < .036). There was no difference in double support time between the PD non-FOG and the other two groups.

A main effect of condition was also found (F(2,88) = 4.32, *P* < .016), which after post hoc analysis confirmed that all participants spent an increased amount of time in double support in the laser condition (0.46 ± 0.27 s) as compared to the ground condition (0.31 ± 0.19 s) (*P* < .022). A trend almost reaching significance was observed that suggested that all participants also spent more time in double support in the laser condition as compared to the baseline condition (Narrow) (0.33 ± 0.53 s) (*P* < .058).

## 4. Discussion

This experiment investigated the influence of visual cues on improving gait of individuals with PD who experience FOG. In recent work, it has been shown that the gait of individuals with PD (whether or not they exhibit FOG) are most affected in a narrow doorway [18]. In the present study, a narrowed doorway was used to specifically evaluate changes in step length, velocity, and step-to-step variability when different visual cues were available. These measures were selected since they have been shown to be closely related to the occurrence of FOG [18]. Overall, the obtained results suggest that the PD FOG group were able to improve and normalize only some of the characteristics of gait (i.e., step length, variability etc.) to that seen in non-FOG individuals with PD and healthy control participants when ground lines were provided. It is important to note that the data reported on in this study was prior to the individuals arriving at the doorway, and so the number of true FOG episodes occurring was not reported. Interestingly, individuals with PD (regardless of whether or not they experienced FOG) did not significantly improve velocity of gait as a result of the ground lines. This is an important point to highlight, since improved step length (with the use of ground lines) would be expected to result in significant improvements to gait velocity. Since this did not occur, it is likely that step timing also contributed (and possibly sacrificed) to improve step length, hence the lack of change in velocity. Several recent works have pointed to a basal ganglia-related timing deficit [19, 20]. Additionally, it is important to note that the laser device was not effective at improving characteristics of gait, and resulted in a further increase in gait variability (as indicated by CV of step time, CV of step length, etc.).

It has been hypothesized that if visual step cues have the ability to improve step length in individuals with PD subject to FOG, then the type of cue presented would be inconsequential, as long as the cue provided dynamic flow information [7]. However, this was not the case in the current study. The benefits gained from the ground lines disappeared with use of the laser device, even though the laser visual cue provided optic flow with similar direction and speed information as normally obtained from the environment. Azulay et al. demonstrated that when dynamic information from visual cues is obstructed through stroboscopic lighting, the benefit gained from visual cues disappear [7]. Their conclusion was that optic flow is the main factor contributing to gait improvement when visual cues are provided. However, it appears that the additional optic flow information of the laser device was a hindrance to these individuals with PD who experience FOG. It has been well documented that an overload of environmental stimuli can lead to gait abnormalities in patients with PD, especially those with documented FOG episodes [10, 13, 21]. Instead of leading to an improvement in gait, the laser device which presented a visual cue in sequence with additional optic flow information may require more attentional resources than the individuals with PD experiencing FOG have at their disposal. This may be one of the reasons that many gait characteristics were negatively influenced by the laser device. 

When used as a visual cue, ground lines resulted in an increased step length. The short shuffling steps which are common in PD and even more debilitating in individuals who experience FOG are being replaced by a more normalized gait pattern through the use of ground lines which is increasingly similar to that observed in the healthy participants. Similar to what has been previously reported [22], both types of visual cues resulted in a decreased cadence as compared to the baseline condition. Also, ground lines improved the increased variability (seen in CV of step time and step length) that was evident in the PD FOG group in the baseline doorway condition (Narrow). This same improvement was not found with use of the laser line device. With use of this cue, the PD FOG group returned to a gait pattern consisting of shorter steps, decreased gait velocity, as well as a slightly further increase in gait variability.

The laser line device led to an increased amount of time spent in double support and within trial step length variability as compared to the ground lines and baseline condition (Narrow). This appears to indicate that participants are experiencing decreased stability, or are attempting to consciously control their gait pattern. The ground lines demonstrated no significant change in any of the gait stability variables as compared to the baseline condition (Narrow). The main differences between these two conditions were that when using the ground lines they had ample time to devise a step pattern before reaching the ground lines. However, with the laser participants faced a time constraint as they were forced to plan individual steps (one at a time) in sequence with the presentation of each laser line. 

It has been previously reported that individuals with PD when presented with visual cues are able to normalize their gait pattern as long as the cues focused their attention on their step pattern [1, 2, 4]. The type of cue presented to the participants was found to be primarily inconsequential. From a practical standpoint, it was hoped that a laser device attached to a participant's waist might replace the need to place lines of tape on the ground, wherever a PD patient might walk. However, in this experiment it was found that those individuals with PD who experience FOG are more selective in the type of cue necessary to improve characteristics of gait. Dynamic visual cues provided by the motorized laser device were not successful in improving the gait of PD FOG. Furthermore, it should be noted that even the ground line condition did not result in a significant improvement in gait velocity regardless of group. Therefore, it appears that when faced with a decreased amount of space (i.e., a doorway) a type of visual cue that can be examined further ahead of time is required in order for gait to be improved. An increase in attention and therefore conscious control of gait is no longer sufficient, but instead preplanned conscious control is required in those individuals with gait disabilities. This information should be taken into consideration when attempting to devise novel methods for prevention of FOG.

Individuals with PD were tested while “on” dopaminergic medication which is a potential limitation of this study, although it is well known that unlike other motor symptoms, freezing is poorly affected by medication [14, 23, 24]. Testing was conducted solely in the “on” state of PD in order to get a true understanding of freezing that may commonly occur in everyday situations. Future studies might include the testing of individuals with FOG while “off” medication in order to obtain a clearer understanding of basal ganglia contributions to freezing. Overall, the results of the current study support the notion that visual cues are beneficial for gait in PD, primarily because they provide information that allows the patient to plan movement in advance. As such, while dynamic flow information may be important, it is not the only factor contributing to gait improvement with visual cues.

## Figures and Tables

**Table 1:** Characteristics of the three groups.

Group	Age-M (years)	Height-M (cm)	UPDRS-M (score)	Years since diagnosed-M (years)	Dose of Levodopa-M (mg)	Gender
PD FOG	72.4 (6.69)	172.51 (8.51)	32.8 (7.34)	9.07 (5.29)	1013.33 (390.27)	13 males, 2 females
PD Non-FOG	72.19 (6.23)	170.66 (9.69)	28.81 (6.35)	5.97 (4.61)	725.0 (449.81)	10 males, 6 females
HC	70.75 (5.98)	167.96 (7.53)	n/a	n/a	n/a	6 males, 10 females

Note*: *M denotes mean, standard deviations found in brackets.

## Abbreviations

PD: Parkinson's disease
FOG: Freezing of gait

## References

[1] Lewis GN, Byblow WD, Walt SE (2000). Stride length regulation in Parkinson’s disease: the use of extrinsic, visual cues. *Brain*.

[2] Morris ME, Iansek R, Matyas TA, Summers JJ (1996). Stride length regulation in Parkinson’s disease: normalization strategies and underlying mechanisms. *Brain*.

[3] Schenkman M (1992). Physical therapy intervention for the ambulatory patient. *Parkinson’s Disease*.

[4] Azulay J-P, Mesure S, Blin O (2006). Influence of visual cues on gait in Parkinson’s disease: contribution to attention or sensory dependence?. *Journal of the Neurological Sciences*.

[5] Rochester L, Hetherington V, Jones D (2005). The effect of external rhythmic cues (auditory and visual) on walking during a functional task in homes of people with Parkinson’s disease. *Archives of Physical Medicine and Rehabilitation*.

[6] van Wegen E, Lim I, de Goede C (2006). The effects of visual rhythms and optic flow on stride patterns of patients with Parkinson’s disease. *Parkinsonism and Related Disorders*.

[7] Azulay J-P, Mesure S, Amblard B, Blin O, Sangla I, Pouget J (1999). Visual control of locomotion in Parkinson’s disease. *Brain*.

[8] Schubert M, Prokop T, Brocke F, Berger W (2005). Visual kinesthesia and locomotion in Parkinson’s disease. *Movement Disorders*.

[9] Bloem BR, Grimbergen YAM, Cramer M, Willemsen M, Zwinderman AH (2001). Prospective assessment of falls in Parkinson’s disease. *Journal of Neurology*.

[10] Okuma Y (2006). Freezing of gait in Parkinson’s disease. *Journal of Neurology*.

[11] Bartels AL, Balash Y, Gurevich T, Schaafsma JD, Hausdorff JM, Giladi N (2003). Relationship between freezing of gait (FOG) and other features of Parkinson’s: FOG is not correlated with bradykinesia. *Journal of Clinical Neuroscience*.

[12] Amboni M, Cozzolino A, Longo K, Picillo M, Barone P (2008). Freezing of gait and executive functions in patients with Parkinson’s disease. *Movement Disorders*.

[13] Schaafsma JD, Balash Y, Gurevich T, Bartels AL, Hausdorff JM, Giladi N (2003). Characterization of freezing of gait subtypes and the response of each to levodopa in Parkinson’s disease. *European Journal of Neurology*.

[14] Nieuwboer A, Giladi N (2008). The challenge of evaluating freezing of gait in patients with Parkinson’s disease. *British Journal of Neurosurgery*.

[15] Kompoliti K, Goetz CG, Leurgans S, Morrissey M, Siegel IM (2000). ’On’ freezing in Parkinson’s disease: resistance to visual cue walking devices. *Movement Disorders*.

[16] Hausdorff JM, Schaafsma JD, Balash Y, Bartels AL, Gurevich T, Giladi N (2003). Impaired regulation of stride variability in Parkinson’s disease subjects with freezing of gait. *Experimental Brain Research*.

[17] Bloem BR, Hausdorff JM, Visser JE, Giladi N (2004). Falls and freezing of Gait in Parkinson’s disease: a review of two interconnected, episodic phenomena. *Movement Disorders*.

[18] Almeida QJ, Lebold CA Freezing of gait in Parkinson’s disease: a perceptual cause for a motor impairment?.

[19] Almeida QJ, Frank JS, Roy EA, Patla AE, Jog MS (2007). Dopaminergic modulation of timing control and variability in the gait of Parkinson’s disease. *Movement Disorders*.

[20] Hausdorff JM, Cudkowicz ME, Firtion R, Wei JY, Goldberger AL (1998). Gait variability and basal ganglia disorders: stride-to-stride variations of gait cycle timing in Parkinson’s disease and Huntington’s disease. *Movement Disorders*.

[21] Nieuwboer A, Dom R, De Weerdt W, Desloovere K, Janssens L, Stijn V (2004). Electromyographic profiles of gait prior to onset of freezing episodes in patients with Parkinson’s disease. *Brain*.

[22] Arias P, Cudeiro J (2008). Effects of rhythmic sensory stimulation (auditory, visual) on gait in Parkinson’s disease patients. *Experimental Brain Research*.

[23] Giladi N, Huber-Mahlin V, Herman T, Hausdorff JM (2007). Freezing of gait in older adults with high level gait disorders: association with impaired executive function. *Journal of Neural Transmission*.

[24] Factor SA, Jennings DL, Molho ES, Marek KL (2002). The natural history of the syndrome of primary progressive freezing gait. *Archives of Neurology*.

